# Impact of Fatty Liver on Acute Pancreatitis Severity

**DOI:** 10.1155/2017/4532320

**Published:** 2017-04-27

**Authors:** Seung Bae Yoon, In Seok Lee, Moon Hyung Choi, Kyungjin Lee, Hyoju Ham, Hyun Jin Oh, Se Hwan Park, Chul-Hyun Lim, Myung-Gyu Choi

**Affiliations:** ^1^Division of Gastroenterology, Department of Internal Medicine, College of Medicine, The Catholic University of Korea, Seoul, Republic of Korea; ^2^Cancer Research Institute, College of Medicine, The Catholic University of Korea, Seoul, Republic of Korea; ^3^Department of Radiology, College of Medicine, The Catholic University of Korea, Seoul, Republic of Korea; ^4^Center for Cancer Prevention and Detection, National Cancer Center, Goyang-si, Republic of Korea

## Abstract

*Aim*. Acute pancreatitis is typically a mild disease, but some patients develop severe courses. Fatty liver changes are seen in patients with acute pancreatitis, but its clinical significance has not been well-studied. We aimed to investigate the relationship between fatty liver and the severity of acute pancreatitis. *Methods*. Unenhanced CT images of patients with acute pancreatitis were retrospectively reviewed by a radiologist, and mean hepatic and splenic attenuation was measured in Hounsfield units (HU). Fatty liver was defined as mean hepatic/splenic HU < 1. *Results*. Among 200 patients, fatty liver was found in 67 (33.5%) and nonfatty liver in 133 (66.5%). Compared with patients without fatty liver, the severity of pancreatitis and levels of serum C-reactive protein were higher in fatty liver patients. The prevalence of local complications, persistent organ failure, and mortality were also higher in patients with fatty liver. Even after adjusting for age, sex, body mass index, and cause of pancreatitis, fatty liver was significantly associated with moderately severe or severe acute pancreatitis. *Conclusions.* Fatty liver may play a prognostic role in acute pancreatitis. Fatty liver could be incorporated into future predictive scoring models.

## 1. Introduction

Acute pancreatitis (AP) is an acute, painful abdominal disease involving the pancreas. Its incidence is on the rise, ranging from 13 to 45 per 100,000 [1]. AP is usually a mild disease, but approximately one-fifth of patients develop severe courses with a mortality rate of 10 to 20% [2–4]. It is important to categorize patients with different severity grades in order to prognosticate and triage, as severe cases require early intensive care and nutritional support. Over recent decades, several clinical, biochemical, and combined scoring models have been developed to assess the prognosis of AP [5, 6]. However, each scoring system has certain shortcomings. For example, the Acute Physiology and Chronic Health Evaluation-II score is complex and the Ranson score is not measured until 48 hours after admission. Serum C-reactive protein (CRP) level has been widely used as a simple prognostic biomarker, but the delay in peak levels makes it less useful on admission [7, 8].

In 2012, the Acute Pancreatitis Classification Working Group modified the Atlanta classification system to improve clinical assessment and treatment of AP [9]. The revised Atlanta classification system focuses mainly on morphologic manifestations by means of computed tomography (CT) [10]. A radiologic scoring system using CT was first introduced by Balthazar et al. in 1985, and a CT severity index with a 10-point scale was developed later [11, 12]. These radiologic scoring systems have been widely used in clinical practice, but they have only shown moderate interobserver agreement [13].

Fatty liver is commonly associated with benign gastrointestinal and pancreaticobiliary diseases, including acute pancreatitis [14]. Fatty liver change, which appears on CT as low attenuation, is frequently detected in AP patients; however, the influence of fatty liver on the severity and clinical outcomes of AP has not been well-studied. A previous study suggested that unenhanced CT demonstrated acceptable levels of sensitivity for detecting hepatic steatosis proved by biopsy [15]. CT scans are usually performed within 72 hours after admission to diagnose and stratify the severity of AP. Therefore, if the finding of fatty liver in an initial CT scan is associated with the severity of AP, it could be useful as an early detectable prognostic marker for in AP. The aim of this study was to investigate the influence of fatty liver on severity and outcomes of AP.

## 2. Materials and Methods

### 2.1. Subjects and Study Design

We retrospectively analyzed patients diagnosed with AP between July 2009 and June 2016 at Seoul St. Mary's Hospital in Seoul, Korea. Diagnosis criteria for AP were defined as the presence of at least 2 of the 3 following factors: (1) abdominal pain characteristic of AP, (2) serum amylase and/or lipase values of more than three times the upper limit of normal, and (3) characteristic findings of AP on CT scan [3]. For patients who were admitted to the hospital for AP more than once during the study period, only the first visit was included in the analysis. Exclusion criteria for the study were AP after endoscopic retrograde cholangiopancreatography, referred cases from other hospitals without an initial CT study, cases without CT scan or unenhanced CT phase, and missing body mass index (BMI) data. The institutional review board approved this study (KC16RISI0585).

### 2.2. Severity Assessment of Acute Pancreatitis

Based on the revised Atlanta classification, AP severity was stratified into three groups: mild, moderately severe, and severe [9]. Mild AP was classified as the absence of organ failure and the absence of local or systemic complications. Moderately severe AP was characterized by the presence of transient organ failure resolving within 48 hours and/or local or systemic complications without persistent organ failure. Severe AP was defined as persistent organ failure lasting more than 48 hours or death. Organ failure was defined as a score ≥ 2 in one of the three assessed organ systems (cardiovascular, respiratory, and renal) using the modified Marshal scoring system [16]. Persistent organ failure was considered if organ failure lasted more than 48 hours. Local complications included acute peripancreatic fluid collection, pancreatic pseudocyst, acute necrotic collection, and walled-off necrosis. Systemic complications were defined as exacerbation of pre-existing comorbid disease, such as coronary artery disease of chronic lung disease, precipitated by the AP. Initial and maximum CRP levels during admission were investigated, and the length of hospital stay was assessed as an outcome parameter of AP.

### 2.3. Measurement of Fatty Liver

**Figure 1:**
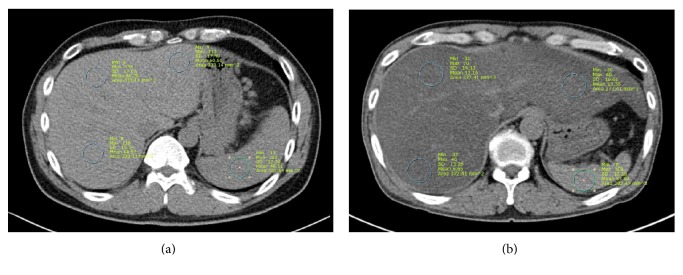
Unenhanced CT images from two patients with acute pancreatitis. (a) 54-year-old man with normal liver attenuation (60 HU) compared with spleen (46 HU). (b) 46-year-old man with severe fatty liver. Mean liver attenuation (13 HU) was significantly lower than spleen attenuation (54 HU).

At our institution, an initial CT scan is usually performed in all AP patients within 72 hours after admission, regardless of AP severity. Unenhanced phase CT images from a picture archiving and communication system were retrospectively reviewed by a radiologist (M.H.C.) blinded to clinical and demographic data. The liver normally has a higher CT attenuation than the spleen. However, fatty liver change leads to a reversal of the liver-to-spleen attenuation ratio [17, 18]. Two consecutive axial CT slices were used to measure the mean Housefield units (HU) for regions of interest (ROIs) in the liver and spleen. ROIs ranged from 200 to 400 mm^2^. In one image, two ROIs were placed in the right hepatic lobe, one ROI in the left hepatic lobe, and one ROI in the spleen (Figure 1). The mean hepatic HU was derived by averaging the HU of all three liver ROIs. The liver-to-spleen attenuation ratio was calculated by dividing the mean hepatic HU by splenic HU. Finally, the results from the two images were averaged. Fatty liver was defined as a liver-to-spleen attenuation ratio of less than 1.

### 2.4. Statistical Analysis

Continuous data are presented as mean ± SD or median (interquartile range), and categorical data are presented as quantities and proportions. Descriptive statistics were used to analyze the baseline characteristics of the study population. Comparisons of characteristics and variables between fatty liver and nonfatty liver groups were performed by using a two-sample independent *t*-test or the Mann-Whitney *U* test for numerical variables and a Pearson chi-square test or the Fisher exact test for nominal variables. Factors potentially associated with moderately severe or severe AP were investigated using logistic regression analysis. The linear-by-linear association method was used for testing trends between fatty liver change and severity of AP in subgroup analyses. Statistical analyses were performed using SPSS 24.0 package (SPSS Inc., Chicago, IL, USA). Statistical significance was defined as *p* < 0.05.

## 3. Results

### 3.1. Baseline Characteristics and Severity of Study Patients

A total of 285 patients were diagnosed with AP during the study period. Of these, 6 patients were transferred from an outside hospital without an early CT study. Two patients did not undergo initial CT scanning in our hospital and 72 patients did not undergo unenhanced phase CT. Five patients had missing data regarding BMI. After excluding these 85 patients, the remaining 200 patients were analyzed.

Baseline characteristics and severity outcomes of study patients (*n* = 200) are shown in Table 1. The study included 119 males (59.5%) and 81 females (40.5%). The mean age was 54.3 ± 17.5 years. The cause of AP was gallstone-related in 72 (36.0%) patients, alcohol abuse in 67 (33.5%) patients, idiopathic in 39 (19.5%) patients, and other in 22 (11.0%) patients. Other causative factors were hypertriglyceridemia (*n* = 6), periampullary tumor (*n* = 6), drugs (*n* = 5), autoimmune-related (*n* = 3), and miscellaneous causes (*n* = 2). According to the revised Atlanta classification, 110 (55.0%) cases were classified as mild, 73 (36.5%) cases as moderately severe, and 17 (8.5%) cases as severe. The incidences of acute peripancreatic fluid collection, pancreatic pseudocyst, acute necrotic collection, and walled-off necrosis were 68 (34.0%), 57 (28.5%), 28 (14.0%), and 14 (7.0%), respectively. Systemic complications and persistent organ failure occurred in 14 (7.0%) and 17 (8.5%) patients, respectively. There were 3 (1.5%) cases of mortality during the study period, and the median (interquartile range) duration of hospitalization was 7 (5–11) days.

### 3.2. Comparison of Characteristics and Severity in AP Patients with and without Fatty Liver

Of the 200 enrolled patients, fatty liver was found in 67 (33.5%) patients.  Table 2 shows a comparison of the baseline characteristics and the severity parameters between patients with and without fatty liver. Mean age was not significantly different between fatty liver and nonfatty liver groups (53.6 ± 16.7 versus 54.7 ± 17.9, *p* = 0.658). Fatty liver was more prevalent in male patients (76.1% versus 51.1%, *p* < 0.001). Mean BMI and the rate of overweight or obese patients were higher in the fatty liver group compared with those in the nonfatty liver group (25.3 ± 4.6 versus 23.4 ± 3.3 and 47.9% versus 25.2%, all *p* = 0.001). Mean arterial pressure, waist circumference, and random glucose level were also higher in the fatty liver group. The prevalence of alcohol-induced pancreatitis was higher in the fatty liver group than that in the nonfatty liver group (44.8% versus 27.8%, *p* = 0.016). There were 19 (28.4%) cases of mild AP, 38 (56.7%) of moderately severe AP, and 10 of (14.9%) severe AP in the fatty liver group, and 91 (68.4%) cases of mild AP, 35 (26.3%) of moderately severe AP, and 7 (5.3%) of severe AP in the nonfatty liver group. The prevalence of mild AP was relatively lower, and the prevalence of moderately severe and severe AP were higher in the fatty liver group compared with that in the nonfatty liver group. Initial and maximum serum CRP levels [median (interquartile range)] of the fatty liver group were significantly higher than those of the nonfatty group [0.9 (0.2–9.6) versus 0.4 (0.1–2.1), *p* = 0.003, and 15.6 (4.1–24.5) versus 4.1 (0.6–14.2), *p* < 0.001, resp.]. The median (interquartile range) duration of hospitalization was 8 (6–13) and 7 (5–10) days in fatty and nonfatty patients, respectively (*p* = 0.057).

### 3.3. Comparison of the Incidence of Complications in AP Patients with and without Fatty Liver

**Figure 2:**
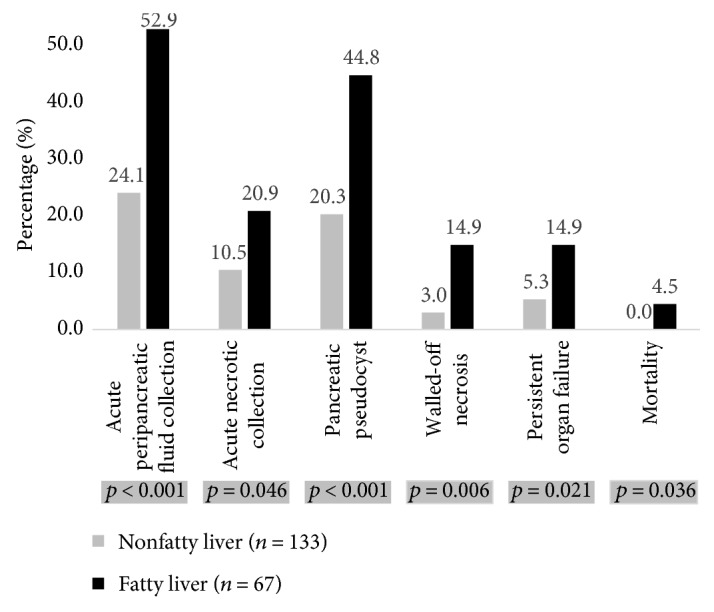
Comparison of complication rates of acute pancreatitis with and without fatty liver.

As shown in Figure 2, a significantly higher proportion of patients with fatty liver had various local or systemic complications compared to nonfatty liver patients. Patients with fatty liver showed higher percentages of acute peripancreatic fluid collection (52.9% versus 24.1%, *p* < 0.001) and acute necrotic collection (20.9% versus 10.5%, *p* = 0.046) than those without fatty liver. Patients with fatty liver also had higher rates of chronic local complications including pancreatic pseudocyst (44.8% versus 20.3%, *p* < 0.001) and walled-off necrosis (14.9% versus 3.0%, *p* = 0.006). Persistent organ failure and mortality rates were also higher in fatty liver patients compared with those in nonfatty liver patients (14.9% versus 5.3%, *p* = 0.021 and 4.5% versus 0.0%, *p* = 0.036, resp.).

### 3.4. Logistic Regression Analysis for Factors Associated With Moderately Severe or Severe AP

Univariate and multivariate logistic regression models for factors associated with moderately severe or severe AP are summarized in Table 3. Univariate analysis showed that fatty liver [odds ratio (OR), 5.47; 95% confidence intervals (CI), 2.83–10.43] was a significant risk factor for moderately severe or severe AP. Even after adjusting for age, sex, BMI, and cause of pancreatitis, fatty liver was significantly associated with moderately severe or severe AP (OR, 4.95; 95% CI, 2.46–9.98). Older age (OR, 1.03; 95% CI, 1.01–1.05) and higher BMI (OR, 1.09; 95% CI, 1.00–1.19) were also risk factors for moderately severe or severe AP. Sex and alcoholic cause were not significantly associated with the severity of AP in our analyses.

### 3.5. Subgroup Analyses

**Figure 3:**
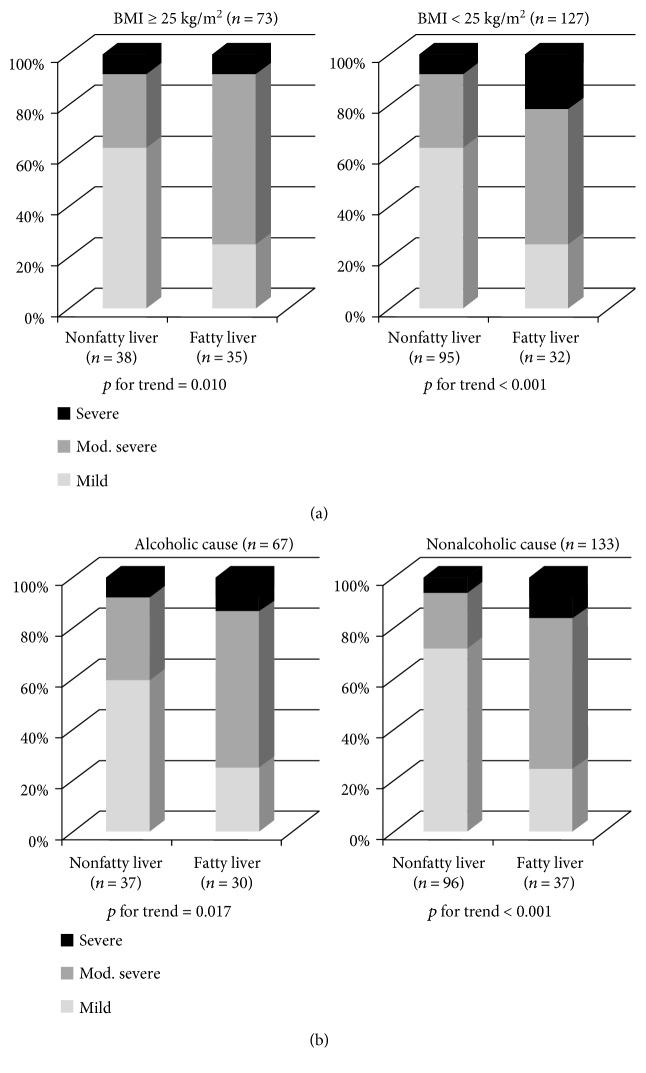
Subgroup analyses on the influence of fatty liver on clinical severity of acute pancreatitis (AP). (a) Fatty liver was associated with severity of AP, in patients with BMI ≥ 25 and BMI < 25. (b) Fatty liver was also associated with severity of AP in patients with alcoholic cause of AP and nonalcoholic cause.

BMI and alcohol abuse are highly associated with fatty liver [19–21]. Therefore, subgroup analyses were done for the categories of BMI (BMI ≥ 25 versus BMI < 25) and cause of pancreatitis (alcoholic versus nonalcoholic cause). Fatty liver was independently associated with severity of AP, both in patients with BMI ≥ 25 and BMI < 25 (*p* for trend = 0.010 and <0.001, resp.; Figure 3). Strong trends in fatty liver and severity of AP were also observed regardless of the cause of pancreatitis (*p* for trend = 0.017 in alcoholic cause and *p* for trend < 0.001 in nonalcoholic cause, resp.).

## 4. Discussion

In this study, we evaluated the influence of fatty liver on severity and clinical outcome in AP. Our results showed that AP patients with fatty liver had more severe clinical features than patients without fatty liver. In patients with AP, fatty liver led to higher rates of local complications, persistent organ failure, and mortality. Fatty liver remained a significant risk factor for severe disease course even after adjusting for multiple confounding factors including age, BMI, and cause of AP.

Fatty liver disease (FLD) is a common hepatic metabolic disorder referring to a wide clinical spectrum ranging from simple hepatic steatosis to severe cirrhosis. According to etiology, fatty liver disease can be classified as alcoholic liver disease and nonalcoholic fatty disease. The prevalence of FLD is rapidly growing due to obesity and alcoholism epidemics, and it affects nearly one-fourth of the general population in both Western and Eastern countries [22, 23]. Beyond damage to the liver, fatty liver can induce diabetes, metabolic syndrome, and atherosclerosis [24–26]. Fatty liver at baseline can be related to higher health care utilization and costs of medical services. Thus, there is concern about FLD increasing [27].

Fatty liver is often seen in AP patients because both diseases share contributing factors such as obesity, alcohol abuse, and hyperlipidemia. In our study, about one-third of patients with AP demonstrated fatty liver change in nonenhanced CT images. Patients with fatty liver showed more severe features and poorer clinical outcomes compared to patients without fatty liver. Interestingly, fatty liver was significantly associated with AP severity regardless of subgroup analysis or adjustment for possible confounding factors, including age and BMI. These findings suggest that fatty liver by itself may be an independent risk factor for severe clinical course of AP. Fatty liver changes in early CT scan can be used as early prognostic markers and can be usefully incorporated into future predictive AP scoring models.

The mechanism for why fatty liver is associated with a more severe course of AP has not been elucidated. The elevation of serum CRP level in the fatty liver group may be one possible explanation for our results. Higher serum CRP levels were consistently found in fatty liver patients compared to controls in previous studies [28, 29]. A chronic proinflammatory state in fatty liver patients may aggravate the course of AP. A recent study suggested that, in rat and human AP models, hepatic steatosis depressed alpha1-antitrypsin levels, which have significant anti-inflammatory properties due to their effect on a wide range of inflammatory cells [30]. Decrease of serum alpha1-antitrypsin levels might lead to excessive activation of inflammation, and this proposed mechanism could support our results. In our study, the difference between initial CRP level in the fatty liver and nonfatty liver group was not great (median, 0.9 versus 0.4); however, the difference in maximum CRP between the two groups was pronounced (median, 15.6 versus 4.1). Serum CRP has been the most widely used biomarker, but the delay in peak levels makes it less useful on admission. Fatty liver in early CT scan can predict the delayed elevation of serum CRP; thus, fatty liver can be a prognostic marker easily determined at initial diagnosis.

In the present study, CT scan was used to assess fatty liver. A previous study suggested that liver and spleen attenuation from unenhanced CT could closely predict the degree of macrovesicular steatosis [15]. Many studies also used the liver-to-spleen attenuation ratio to define the fatty liver change [17, 18]. CT scanning is a clinically suitable method for the assessment of fatty liver in AP patients, and contrast-enhanced CT is the primary tool used to assess and monitor AP. Moreover, the importance of CT has increased in patients with AP because the revised Atlanta classification system is based mainly on morphologic features. Combined unenhanced and enhanced CT scans may be useful in assessing the status of both fatty liver and AP.

On retrospective analysis, this study had some limitations. First, we acknowledge the limitation of excluding approximately one-fourth of patients who did not undergo unenhanced phase CT scan. The role of unenhanced CT imaging had not been established before our study, and the unenhanced phase has not been routinely performed in AP patients. Second, we could not exclude patients taking medications that could cause liver steatosis or could not consider the effects of such drugs. Third, the proportion of idiopathic AP in our study (19.5%) was slightly higher than that generally known. Unfortunately, we could not evaluate genetic predisposition or sphincter of odd dysfunction as the cause of unexplained pancreatitis. In addition, endoscopic ultrasound or magnetic resonance cholangiopancreatography was not routinely performed in our patients with idiopathic AP. For these reasons, the rate of idiopathic AP would have been overestimated. Finally, pro- and anti-inflammatory cytokine levels were not examined, and the mechanism by which fatty liver aggravates AP remains to be defined. Further studies including cytokine data are needed to clarify the role of fatty liver in patients with AP.

In conclusion, fatty liver was frequently detected by unenhanced CT scan and was strongly correlated with AP severity. Fatty liver led to higher rates of local complications, persistent organ failure, and mortality in AP. This simple grading system is a potentially valuable prognostic marker that should be considered for use in future predictive scoring systems for AP.

## Figures and Tables

**Table 1:** Baseline characteristics and severity outcomes of study patients (*n* = 200).

Patient characteristics	
Age, mean ± SD (range), years	54.3 ± 17.5 (22–87)
Sex, male (%)	119 (59.5%)
Etiology	
Gallstone-related	72 (36.0%)
Alcohol abuse	67 (33.5%)
Idiopathic	39 (19.5%)
Other	22 (11.0%)
Severity outcome	
Revised Atlanta classification	
Mild	110 (55.0%)
Moderately severe	73 (36.5%)
Severe	17 (8.5%)
Peripancreatic fluid collection	68 (34.0%)
Pancreatic pseudocyst	57 (28.5%)
Acute necrotic collection	28 (14.0%)
Walled-off necrosis	14 (7.0%)
Systemic complications	14 (7.0%)
Persistent organ failure	17 (8.5%)
Mortality	3 (1.5%)
Duration of hospitalization, median (IQR), days	7 (5–11)

IQR: interquartile range.

**Table 2:** Comparison of characteristics and severity parameters between AP patients with and without fatty liver.

Parameters	Fatty liver (*n* = 67)	Nonfatty liver (*n* = 133)	*p*
Age, mean ± SD, years	53.6 ± 16.7	54.7 ± 17.9	0.658
Sex, male (%)	51 (76.1%)	68 (51.1%)	<0.001
Body mass index, kg/m^2^	25.3 ± 4.6	23.4 ± 3.3	0.001
Overweight or obese (BMI ≥ 25 kg/m^2^, %)	35 (47.9%)	32 (25.2%)	0.001
Mean arterial pressure, mm Hg	94.8 ± 14.3	90.7 ± 12.7	0.038
Waist circumference, cm	90.7 ± 10.0	83.3 ± 8.8	<0.001
Random blood glucose, mg/dL	179.2 ± 66.2	150.4 ± 71.0	0.006
Serum triglyceride, mg/dL	224.9 ± 312.4	159.2 ± 190.9	0.123
Alcohol-induced pancreatitis (%)	30 (44.8%)	37 (27.8%)	0.016
Severity by revised Atlanta classification (%)			<0.001
Mild	19 (28.4%)	91 (68.4%)	
Moderately severe	38 (56.7%)	35 (26.3%)	
Severe	10 (14.9%)	7 (5.3%)	
Initial serum CRP level, median (IQR), mg/dL^∗^	0.9 (0.2–9.6)	0.4 (0.1–2.1)	0.003
Maximum serum CRP level, median (IQR), mg/dL^∗^	15.6 (4.1–24.5)	4.1 (0.6–14.2)	<0.001
Duration of hospitalization, median (IQR), days	8 (6–13)	7 (5–10)	0.057

AP: acute pancreatitis; CRP: C-reactive protein; IQR: interquartile range; ^∗^normal range of CRP: 0–0.3 mg/dL.

**Table 3:** Logistic regression analysis for factors associated with moderately severe or severe acute pancreatitis.

Factor	Univariate analysis		Multivariate analysis	
	OR	95% CI	OR	95% CI
Fatty liver versus nonfatty liver	5.47^∗^	2.87–10.43	4.95^∗^	2.46–9.98
Age	1.02^∗^	1.00–1.04	1.03^∗^	1.01–1.05
Male versus female	1.34	0.76–2.37	0.86	0.42–1.75
BMI	1.11^∗^	1.03–1.20	1.09^∗^	1.00-1.19
Alcoholic cause versus nonalcoholic cause	1.55	0.86–2.80	2.07	0.96–4.45

CI: confidence intervals; OR: odds ratio; ∗ indicates *p* value of <0.05.
